# *BMC Medical Informatics and Decision Making* reviewer acknowledgement 2014

**DOI:** 10.1186/s12911-015-0135-9

**Published:** 2015-03-05

**Authors:** Giulia Mangiameli

**Affiliations:** BioMed Central, Floor 6, 236 Gray’s Inn Road, London, WC1X 8HB United Kingdom

## Abstract

The editors of BMC Medical Informatics and Decision Making would like to thank all our reviewers who have contributed to the journal in Volume 14 (2014).

**Jos Aarts**

Netherlands

**Samir Abdelrahman**

USA

**Dionisio Acosta**

UK

**Ann Adams**

UK

**Danielle Albers**

USA

**Dana Alden**

USA

**Larry Allen**

USA

**Robert Anderson**

USA

**Carl Asche**

USA

**Nega Assefa**

Ethiopia

**Knut Magne Augestad**

Norway

**Dirk Avonts**

Belgium

**Steven Babin**

USA

**Richard Bache**

UK

**Nick Bansback**

Canada

**Elissa Bantug**

USA

**Paul Barr**

USA

**Jennifer Barton**

USA

**Andrew Bateman**

UK

**Hilary Bekker**

UK

**Brian Bell**

UK

**Paul Bentley**

UK

**Solomon Berhe**

USA

**Simon Bernard**

France

**Jit Biswas**

Singapore

**Joaquin Blaya**

USA

**David Blazes**

USA

**Marcus Bloice**

Austria

**Jennifer Blumenthal-Barby**

USA

**Raymond Bond**

UK

**Diego Bosca Tomas**

Spain

**Claus Bossen**

Denmark

**Erin Bowles**

USA

**Teresa Brady**

USA

**Hillel Braude**

Israel

**Sylvia Brinkman**

Netherlands

**Benjamin Brown**

UK

**Natale Daniele Brunetti**

Italy

**Christopher Buckingham**

UK

**Anna L Buczak**

USA

**Howard Burkom**

USA

**Aidan Byrne**

UK

**Dexter Canoy**

UK

**Ewart Carson**

UK

**Víctor H. Castillo**

Mexico

**Leo Anthony Celi**

USA

**Emmanuel Chazard**

France

**Chi-Yuan Chen**

Taiwan

**Shih-Chih Chen**

Taiwan

**Tain-Junn Cheng**

Taiwan

**Jean-Paul Chretien**

USA

**Cynthia Chuang**

USA

**Imre Cikajlo**

Slovenia

**James Cimino**

USA

**Karen Clark**

USA

**Mark Clements**

Sweden

**Amy Coenen**

USA

**Heather Colquhoun**

Canada

**Elizabeth Comino**

Australia

**Kathrin Cresswell**

UK

**Ricardo Cruz-Correia**

Portugal

**Gianfranco Damiani**

Italy

**Berry De Bruijn**

Canada

**Jeroen De Bruin**

Austria

**Dylan De Lange**

Netherlands

**Paula De Toledo**

Spain

**Filip De Turck**

Belgium

**Ken Deal**

Canada

**Rob Deardon**

Canada

**Marie-Dominique Devignes**

France

**Haryana Dhillon**

Australia

**Brad Dicianno**

USA

**Joerg Dirmaier**

Germany

**Brian Dixon**

USA

**Benjamin Djulbegovic**

USA

**James Dolan**

USA

**Hailong Dong**

China

**Heather Douglas**

Australia

**Tobias Dreischulte**

UK

**Georg Duftschmid**

Austria

**Marie-Anne Durand**

UK

**Jonathan Dushoff**

Canada

**Claudio Eccher**

Italy

**Adrian Edwards**

UK

**Tanja Effing**

Australia

**Emma Eggleston**

UK

**Aboozar Eghdam**

Sweden

**Birgit Eiermann**

Sweden

**Eric Eisenste**

USA

**Yevgeniy Elbert**

USA

**Mortada El-Shabrawi**

Egypt

**Sonya Eremenco**

USA

**Jim Fackler**

USA

**Morten Wang Fagerland**

Norway

**Kathleen Fairfield**

USA

**Miguel Fernández**

Spain

**Jesualdo Tomas Fernandez-Breis**

Spain

**Carlos Fernandez-Llatas**

Spain

**Christopher Fillmore**

USA

**Michael Fischer**

USA

**Jesus Fontecha**

Spain

**Uno Fors**

Sweden

**Dimitrios Fotiadis**

Greece

**Paolo Fraccaro**

UK

**Hamish Fraser**

UK

**Hendrik Friederichs**

Germany

**Dominick Frosch**

USA

**Elies Fuster-Garcia**

Spain

**Anna Gagliardi**

Canada

**Helga Gardarsdottir**

Netherlands

**Mark Garner**

UK

**Oscar Garza**

USA

**Becky Genberg**

USA

**Paul George**

USA

**Andrew Georgiou**

Australia

**Karthik Ghosh**

USA

**Matthias Gietzelt**

Germany

**Anik Giguere**

Canada

**Francisco Gimenez**

USA

**Michael Gionfriddo**

USA

**Michael Gleicher**

USA

**William Goggins**

Hong Kong

**Yang Gong**

USA

**William Goossen**

Netherlands

**Kathleen Gray**

Australia

**Joanne Greenhalgh**

UK

**Wilfried Grossmann**

Austria

**Cyril Grouin**

France

**Yulong Gu**

New Zealand

**Freedom Nkhululeko Gumedze**

South Africa

**Werner O Hackl**

Austria

**David Hanauer**

USA

**Sebastien Haneuse**

USA

**Lyn Hanmer**

South Africa

**Terry John Hannan**

Australia

**Yuji Hatanaka**

Japan

**Brett Hauber**

USA

**Jan A Hazelzet**

Netherlands

**Jennifer Hefner**

USA

**David Hepner**

USA

**William Hersh**

USA

**Harry Hochheiser**

USA

**Jason Hockenberry**

USA

**Wolfgang Hoffmann**

Germany

**Anne Holland**

Australia

**Tim Holt**

UK

**Marcus Horstmann**

Germany

**Susan Hrisos**

UK

**Zhengxing Huang**

China

**Amelia Huck**

USA

**Linda Huibers**

Netherlands

**Vojtech Huser**

USA

**Yun Jang**

South Korea

**Caroline Jay**

UK

**Namita Jayaprakash**

USA

**Masahito Jimbo**

USA

**Constance Johnson**

USA

**Stan Kachnowski**

USA

**David Kaelber**

USA

**Devan Kansagara**

USA

**Judith Katzenellenbogen**

Australia

**Justin Keen**

UK

**Ron Keizer**

Sweden

**Nicole Ketelaar**

Netherlands

**Do Han Kim**

South Korea

**Patricia Kipnis**

USA

**Eric Kirkendall**

USA

**Jeffrey Klann**

USA

**Guilan Kong**

China

**Petros Kountouris**

Cyprus

**Vassilis Koutkias**

France

**Sunil Kripalani**

USA

**Suman Kumar**

USA

**Zach Landis-Lewis**

USA

**Giordano Lanzola**

Italy

**Gunter Laux**

Germany

**France Légaré**

Canada

**Morgan Levine**

USA

**Kent Lewandrowski**

USA

**Linda Li**

Canada

**Qin Li**

USA

**Jialiang Li**

Singapore

**Valentina Lichtner**

UK

**Chad Lin**

Australia

**Andreas Linninger**

USA

**Ellen Lipstein**

USA

**Gil L'Italien**

USA

**Chiung-Ju Liu**

USA

**Robert Logan**

USA

**Corrado Loglisci**

Italy

**Carl Lombard**

South Africa

**Gang Luo**

USA

**Jake Luo**

USA

**Yuan Luo**

USA

**Giovanni Maccioni**

Italy

**Stephen Madden**

Ireland

**Nicos Maglaveras**

Greece

**Jose Alberto Maldonado**

Spain

**Katherine Mallin**

USA

**Micha Mandel**

Israel

**John Mantas**

Greece

**Borja Martínez-Pérez**

Spain

**Keith Mcinnes**

USA

**Robert Mcleod**

Canada

**Jorit Meesters**

Netherlands

**Genevieve Melton**

USA

**William Meurer**

USA

**Silvia Miksch**

Austria

**Jeremy Miles**

UK

**Laura Militello**

USA

**Riccardo Miotto**

USA

**Arnold Mitnitski**

Canada

**David Moner**

Spain

**Muthu Rama Krishnan Mookiah**

Singapore

**Andrew Mooney**

UK

**Markus Muellner**

Austria

**Hillary Mull**

USA

**Hussain Mulla**

UK

**Adolfo Muñoz Carrero**

Spain

**Jeannette Murphy**

UK

**Amir-Homayoon Najmi**

USA

**Christos Nakas**

Greece

**Guillermo Navarro-Arribas**

Spain

**Goran Nenadic**

UK

**Mathieu Nendaz**

Switzerland

**Chin Fen Neoh**

Malaysia

**Wendy Nilsen**

USA

**Mikael Nystrom**

Sweden

**Elke Ochsmann**

Germany

**Margaret Olsen**

USA

**Crsitina Oroviogoicoechea**

Spain

**Lucinda Orsini**

USA

**Casey Overby**

USA

**Claudia Pagliari**

UK

**Carlos Luis Parra**

Spain

**Beverley Paterson**

Australia

**Stephen Pauker**

USA

**Julie Pavlin**

USA

**Philip Payne**

USA

**Christopher Pearce**

Australia

**Niels Peek**

Netherlands

**Jennifer Pellowski**

USA

**Javier Pereira**

Spain

**Adler Perotte**

USA

**Ruth Perou**

USA

**Dimitra Petrakaki**

UK

**David Petrie**

Canada

**Shobha Phansalkar**

USA

**Victoria Phillips**

USA

**Christoph Pimmer**

Switzerland

**Michael Pine**

USA

**Bruno Piotti**

Italy

**Catherine Plaisant**

USA

**Yasmine Probst**

Australia

**Silvana Quaglini**

Italy

**Jialan Que**

USA

**Arvind Raghu**

UK

**Liane Ramac-Thomas**

USA

**Jan Ramon**

Belgium

**Mark Rapoport**

Canada

**Theo Raynor**

UK

**Rachel Richesson**

USA

**Christoph Rinner**

Austria

**Dan Robotham**

UK

**Pedro Pereira Rodrigues**

Portugal

**Charo Rodriguez**

Canada

**Arne Roets**

Belgium

**Bjorn Rosengren**

Sweden

**Carolyn Rutter**

USA

**Didier Saey**

Canada

**Laura Sahm**

Ireland

**Satya Sahoo**

USA

**Glenn Salkeld**

Australia

**Nancy Santesso**

Canada

**Isabelle Scholl**

Germany

**Stefan Schulz**

Germany

**Martin Sedlmayr**

Germany

**Karen Sepucha**

USA

**Merlijn Sevenster**

USA

**Jill Shawe**

UK

**Yao Shieh**

Taiwan

**Ronald Siebes**

Netherlands

**Magenta Simmons**

Australia

**Sonal Singh**

USA

**Peter Singleton**

UK

**Sam Smith**

UK

**Vernon Smith**

USA

**Janet Smylie**

Canada

**Changhong Song**

USA

**Emmanouil Spanakis**

Greece

**Matthew Sperrin**

UK

**Alistair Stewart**

New Zealand

**Michael Stoto**

USA

**Karl Stroetmann**

Denmark

**Fay Strohschein**

Canada

**Sandeep Subramanian**

Canada

**Walton Sumner**

USA

**Gautham Suresh**

USA

**Maria Taboada**

Spain

**Amirhossein Takian**

UK

**Xin Tang**

USA

**Cui Tao**

USA

**Lee Taylor**

Australia

**Paul Taylor**

UK

**Marta Terrile**

Italy

**Carl Thompson**

UK

**William Thompson**

USA

**Kamala Thriemer**

Australia

**Thankam Thyvalikakath**

USA

**Hao Tian**

USA

**Lili Tian**

USA

**Jennifer Tieman**

Australia

**Brian Tiplady**

UK

**Luca Toldo**

Germany

**Christian Tominski**

Germany

**Salvador Tortajada**

Spain

**Lyndal Trevena**

Australia

**Manolis Tsiknakis**

Greece

**Allan Tucker**

UK

**Kim Unertl**

USA

**Mark Unruh**

USA

**Ben Van Calster**

Belgium

**Moniek Van De Luijtgaarden**

Netherlands

**Geert Van Der Heijden**

Netherlands

**Mia Van Der Kop**

Cameroon

**Mariette Van Engen-Verheul**

Netherlands

**Mark Van Grinsven**

Netherlands

**Maaike Van Mourik**

Netherlands

**Peter Van Ooijen**

Netherlands

**Tibor Van Rooij**

Canada

**Marina Velikova**

Netherlands

**Joshua Vest**

USA

**Robert Volk**

USA

**Miriam Vollenbroek-Hutten**

Netherlands

**Jana Vranova**

Czech Republic

**Bernand Vrijens**

Belgium

**Sholom Wacholder**

USA

**Amy Waller**

Australia

**Colin Walsh**

USA

**Jing Wang**

USA

**Shengyao Wang**

China

**Patrick Waterson**

UK

**Griffin Weber**

USA

**Wei Wei**

USA

**Wei-Qi Wei**

USA

**Charlene Weir**

USA

**Brandon Welch**

USA

**Jobke Wentzel**

Netherlands

**James Whyte**

USA

**Lisa Susan Wieland**

USA

**Janice Wiley**

Australia

**David Wiljer**

Canada

**Holly Witteman**

Canada

**Til Wykes**

UK

**Guohui Xiao**

Italy

**Hua Xu**

USA

**Lingzhong Xu**

China

**Zhiheng Xu**

USA

**Che-Ming Yang**

Taiwan

**Ying Yang**

USA

**Ping Yu**

Australia

**Mike Zender**

USA

**Weiya Zhang**

UK

**Zhen Zhang**

USA

**Brian Zikmund-Fisher**

USA

**Patricia Zrelak**

USA

**Paola Zucchi**

Brazil

